# 2-Bromo-4-chloro-6-[(2,6-diisopropyl­phen­yl)imino­meth­yl]phenol

**DOI:** 10.1107/S1600536808035071

**Published:** 2008-11-08

**Authors:** K. Kanmani Raja, I. Mohammed Bilal, S. Thambidurai, G. Rajagopal, A. SubbiahPandi

**Affiliations:** a Department of Chemistry, BSA Crescent Engineering College, Chennai 600 048, India; b Department of Chemistry, Periyare Arts College, Cuddalore 607 001, India; c Department of Chemistry, Government Arts College (Men) (Autonomous), Nandanam, Chennai 600 035, India; d Department of Physics, Presidency College (Autonomous), Chennai 600 005, India

## Abstract

There are two molecules in the asymmetric unit of the title compound, C_19_H_21_BrClNO, with dihedral angles between the aromatic rings of 70.0 (2) and 81.9 (3)°. The crystal structure is stabilized by inter­molecular C—H⋯π and C—Br⋯π inter­actions. In additional, the stacked mol­ecules exhibit intra­molecular O—H⋯N hydrogen bonds.

## Related literature

For the synthesis, see: Chang *et al.* (1998 ▶). For Schiff base compounds in coordination chemistry, see: Pu (2008 ▶). For Schiff base compounds containing salicyl­idene, see: Figuet *et al.* (2001 ▶); Kennedy & Reglinski (2001 ▶); Thamotharan *et al.* (2003 ▶). For related structures, see: Lin *et al.* (2005 ▶); Chen & Ye (2008 ▶).
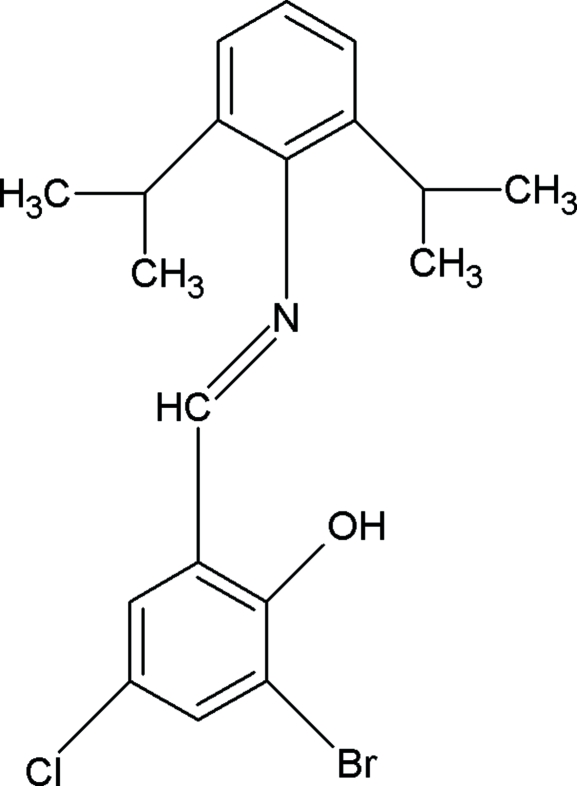


## Experimental

### 

#### Crystal data

C_19_H_21_BrClNO
*M* *_r_* = 394.73
Monoclinic, 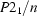

*a* = 11.356 (2) Å
*b* = 15.045 (3) Å
*c* = 22.660 (5) Å
β = 91.36 (3)°
*V* = 3870.4 (13) Å^3^
*Z* = 8
Mo *K*α radiation
μ = 2.27 mm^−1^
*T* = 293 (2) K
0.26 × 0.15 × 0.15 mm

#### Data collection

Bruker APEXII CCD area-detector diffractometer
Absorption correction: multi-scan (*SADABS*; Sheldrick, 1996 ▶) *T*_min_ = 0.672, *T*_max_ = 0.712
36408 measured reflections
6820 independent reflections
4111 reflections with *I* > 2σ(*I*)
*R*_int_ = 0.045

#### Refinement

*R*[*F* ^2^ > 2σ(*F* ^2^)] = 0.045
*wR*(*F* ^2^) = 0.146
*S* = 1.04
6820 reflections
424 parameters
H-atom parameters constrained
Δρ_max_ = 0.45 e Å^−3^
Δρ_min_ = −0.60 e Å^−3^

### 

Data collection: *APEX2* (Bruker, 2004 ▶); cell refinement: *APEX2* and *SAINT* (Bruker, 2004 ▶); data reduction: *SAINT*; program(s) used to solve structure: *SHELXS97* (Sheldrick, 2008 ▶); program(s) used to refine structure: *SHELXL97* (Sheldrick, 2008 ▶); molecular graphics: *ORTEP-3* (Farrugia, (1997 ▶); software used to prepare material for publication: *SHELXL97* and *PLATON* (Spek, 2003 ▶).

## Supplementary Material

Crystal structure: contains datablocks global, I. DOI: 10.1107/S1600536808035071/lx2070sup1.cif

Structure factors: contains datablocks I. DOI: 10.1107/S1600536808035071/lx2070Isup2.hkl

Additional supplementary materials:  crystallographic information; 3D view; checkCIF report

## Figures and Tables

**Table 1:** Hydrogen-bond geometry (Å, °)

*D*—H⋯*A*	*D*—H	H⋯*A*	*D*⋯*A*	*D*—H⋯*A*
O1—H1*A*⋯N1	0.82	1.87	2.598 (4)	147
O2—H2*A*⋯N2	0.82	1.88	2.610 (4)	147
C28—H28*A*⋯*Cg*1^i^	0.96	2.96	3.773 (6)	144
C16—Br1⋯*Cg*4^i^	1.88	3.53	4.75 (2)	120

Symmetry code: (i) 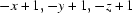. *Cg*1 is the centroid of the C14–C19 benzene ring and *Cg*4 is the centroid of the C33–C38 benzene ring..

**Fig. 1.**
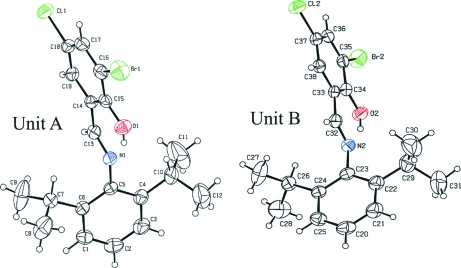
The molecular structure of title compound showing 30% probability displacement ellipsoids.

**Fig. 2.**
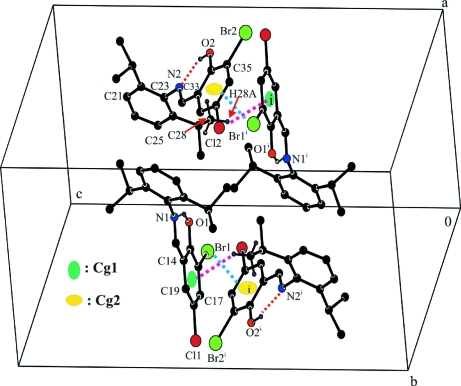
C—H···π, C—Br···π and O—H···N interactions (dotted lines) in the title compound. Cg denotes the ring centroid. [Symmetry code: (i) -x+1, -y+1, -z+1.]

## supplementary crystallographic information

### Comment

Schiff base compounds have been of great interest for many years. These compounds play an important role in the development of coordination chemistry related to catalysis and enzymatic reactions, magnetism and molecular architectures (Pu, 2008). As a part of our ongoing investigation in this field we have determined the crystal structure of the title compound, (I).

Fig.1 shows the asymmetric unit consisting of two molecules of (I) viz. unit A and unit B. The two crystallographically independent molecules have the same geometrical parameters within the precision of the experiments. The bond lengths and angles in (I) are comparable to the corresponding values in the related structure, 2-Bezyliminomethyl-6-bromo-4-chloro-phenol (Pu, 2008). Like other Schiff base compounds containing salicylidene (Figuet *et al.*, 2001; Kennedy & Reglinski, 2001; Thamotharan *et al.*, 2003) the hydroxyl groups form intramolecular hydrogen bonds with the N atoms, thereby completing six-membered rings (Fig. 2). The molecular packing is stabilized by intermolecular C—H···π and C—Br···π interactions, with a C28—H28A···*Cg*1^i^ separation of 2.96 Å and a C16—Br1···*Cg*2^i^ separation of 3.532 (5) Å (Fig. 2 and Table 1; *Cg*1 and *Cg*2 are the centroids of the C14-C19 and C33-C38 benzene rings, respectively,  symmetry code as in Fig. 2). In addition, the molecular packing is further stabilized by two intramolecular O—H···N hydrogen bonds (Table 1).

### Experimental

The title compound was synthesized by refluxing an ethanol solution (20 ml) of 5-bromo-3-chloro-2-hydroxybenzaldehyde (1.72 g, 10 mmol) and 2,6-diisopropylaniline (1.72 g, 10 mmol), at 80°C for 2 h. Upon cooling to 0°C, a yellow solid crystalline product was obtained. The precipitate was filtered off and washed with cold ethanol. Single crystal of good diffraction quality was obtained by the recrystallization of compound with ethanol solution by slow evaporation method.

### Refinement

All H atoms were fixed geometrically and allowed to ride on their parent C atoms, with C—H distances fixed in the range (0.82–0.97)Å with *U*_iso_(H)= 1.5*U*_eq_(methyl H) and 1.2*U*_eq_(for other H atoms).

### Crystal data

**Table:**

C_19_H_21_BrClNO	*F*_000_ = 1616
*M**_r_* = 394.73	*D*_x_ = 1.355 Mg m^−^^3^
Monoclinic, *P*2_1_/*n*	Mo *K*α radiation λ = 0.71073 Å
Hall symbol: -P 2yn	Cell parameters from 9432 reflections
*a* = 11.356 (2) Å	θ = 1.6–28.1º
*b* = 15.045 (3) Å	µ = 2.27 mm^−^^1^
*c* = 22.660 (5) Å	*T* = 293 (2) K
β = 91.36 (3)º	Block, colourless
*V* = 3870.4 (13) Å^3^	0.26 × 0.15 × 0.15 mm
*Z* = 8	

### Data collection

**Table:**

Bruker APEXII CCD area-detector diffractometer	6820 independent reflections
Radiation source: fine-focus sealed tube	4111 reflections with *I* > 2σ(*I*)
Monochromator: graphite	*R*_int_ = 0.045
Detector resolution: 10.0 pixels mm^-1^	θ_max_ = 25.0º
*T* = 293(2) K	θ_min_ = 1.6º
ω and φ scans	*h* = −13→13
Absorption correction: Multi-scan(SADABS; Sheldrick, 1996)	*k* = −17→17
*T*_min_ = 0.672, *T*_max_ = 0.712	*l* = −26→26
36408 measured reflections	

### Refinement

**Table:**

Refinement on *F*^2^	Hydrogen site location: difference Fourier map
Least-squares matrix: full	H-atom parameters constrained
*R*[*F*^2^ > 2σ(*F*^2^)] = 0.045	*w* = 1/[σ^2^(*F*_o_^2^) + (0.0678*P*)^2^ + 2.2687*P*] where *P* = (*F*_o_^2^ + 2*F*_c_^2^)/3
*wR*(*F*^2^) = 0.146	(Δ/σ)_max_ < 0.001
*S* = 1.04	Δρ_max_ = 0.45 e Å^−^^3^
6820 reflections	Δρ_min_ = −0.60 e Å^−^^3^
424 parameters	Extinction correction: SHELXL97 (Sheldrick, 2008), Fc^*^=kFc[1+0.001xFc^2^λ^3^/sin(2θ)]^-1/4^
Primary atom site location: structure-invariant direct methods	Extinction coefficient: 0.0011 (2)
Secondary atom site location: difference Fourier map	

### Special details

**Table:**

Geometry. All esds (except the esd in the dihedral angle between two l.s. planes) are estimated using the full covariance matrix. The cell esds are taken into account individually in the estimation of esds in distances, angles and torsion angles; correlations between esds in cell parameters are only used when they are defined by crystal symmetry. An approximate (isotropic) treatment of cell esds is used for estimating esds involving l.s. planes.
Refinement. Refinement of F^2^ against ALL reflections. The weighted R-factor wR and goodness of fit S are based on F^2^, conventional R-factors R are based on F, with F set to zero for negative F^2^. The threshold expression of F^2^ > 2sigma(F^2^) is used only for calculating R-factors(gt) etc. and is not relevant to the choice of reflections for refinement. R-factors based on F^2^ are statistically about twice as large as those based on F, and R- factors based on ALL data will be even larger.

### Fractional atomic coordinates and isotropic or equivalent isotropic displacement parameters (Å^2^)

**Table:**

	*x*	*y*	*z*	*U*_iso_*/*U*_eq_	
Br1	0.40344 (6)	0.77524 (4)	0.52632 (2)	0.0958 (2)	
Br2	0.84887 (5)	−0.02832 (4)	0.51695 (3)	0.0954 (2)	
Cl1	−0.06647 (12)	0.75082 (9)	0.58127 (6)	0.0945 (5)	
Cl2	0.39884 (10)	0.00891 (9)	0.60014 (6)	0.0805 (4)	
O1	0.4129 (2)	0.60711 (19)	0.59597 (13)	0.0682 (8)	
H1A	0.4147	0.5609	0.6152	0.102*	
O2	0.8822 (2)	0.14048 (19)	0.58425 (13)	0.0672 (8)	
H2A	0.8905	0.1875	0.6022	0.101*	
N1	0.3289 (3)	0.4748 (2)	0.65542 (13)	0.0535 (8)	
N2	0.8189 (3)	0.2768 (2)	0.64732 (14)	0.0564 (8)	
C1	0.3129 (4)	0.2336 (3)	0.68334 (19)	0.0645 (11)	
H1	0.2833	0.1820	0.6660	0.077*	
C2	0.3718 (4)	0.2290 (3)	0.7371 (2)	0.0744 (13)	
H2	0.3799	0.1745	0.7561	0.089*	
C3	0.4184 (4)	0.3039 (3)	0.76285 (19)	0.0748 (13)	
H3	0.4600	0.2991	0.7986	0.090*	
C4	0.4050 (4)	0.3865 (3)	0.73692 (17)	0.0624 (11)	
C5	0.3417 (3)	0.3900 (3)	0.68311 (16)	0.0499 (9)	
C6	0.2973 (3)	0.3146 (3)	0.65476 (16)	0.0512 (9)	
C7	0.2381 (4)	0.3171 (3)	0.59384 (19)	0.0658 (11)	
H7	0.2328	0.3797	0.5821	0.079*	
C8	0.3125 (5)	0.2709 (5)	0.5489 (2)	0.118 (2)	
H8A	0.3185	0.2089	0.5585	0.178*	
H8B	0.3897	0.2969	0.5493	0.178*	
H8C	0.2766	0.2776	0.5104	0.178*	
C9	0.1148 (5)	0.2808 (6)	0.5929 (3)	0.145 (3)	
H9A	0.1171	0.2179	0.5997	0.218*	
H9B	0.0779	0.2925	0.5551	0.218*	
H9C	0.0705	0.3090	0.6232	0.218*	
C10	0.4545 (5)	0.4695 (3)	0.7655 (2)	0.0890 (16)	
H10	0.4494	0.5173	0.7363	0.107*	
C11	0.3828 (7)	0.4963 (6)	0.8163 (4)	0.205 (5)	
H11A	0.3029	0.5062	0.8032	0.307*	
H11B	0.4142	0.5500	0.8333	0.307*	
H11C	0.3847	0.4500	0.8455	0.307*	
C12	0.5830 (6)	0.4609 (5)	0.7853 (3)	0.136 (3)	
H12A	0.6164	0.5190	0.7906	0.204*	
H12B	0.6256	0.4293	0.7559	0.204*	
H12C	0.5879	0.4289	0.8220	0.204*	
C13	0.2279 (4)	0.5113 (3)	0.65150 (15)	0.0518 (10)	
H13	0.1642	0.4836	0.6688	0.062*	
C14	0.2094 (3)	0.5947 (2)	0.62079 (15)	0.0494 (9)	
C15	0.3027 (4)	0.6388 (2)	0.59373 (16)	0.0522 (10)	
C16	0.2787 (4)	0.7172 (3)	0.56345 (17)	0.0627 (11)	
C17	0.1650 (5)	0.7516 (3)	0.55948 (18)	0.0703 (13)	
H17	0.1500	0.8044	0.5393	0.084*	
C18	0.0757 (4)	0.7064 (3)	0.58573 (19)	0.0657 (12)	
C19	0.0958 (4)	0.6301 (3)	0.61582 (17)	0.0579 (10)	
H19	0.0336	0.6010	0.6334	0.070*	
C20	0.8777 (5)	0.5253 (3)	0.7251 (2)	0.0784 (14)	
H20	0.8885	0.5804	0.7430	0.094*	
C21	0.9117 (4)	0.4493 (4)	0.7540 (2)	0.0772 (14)	
H21	0.9476	0.4540	0.7912	0.093*	
C22	0.8946 (4)	0.3656 (3)	0.72966 (18)	0.0672 (12)	
C23	0.8386 (3)	0.3623 (3)	0.67371 (17)	0.0538 (10)	
C24	0.8085 (3)	0.4385 (3)	0.64220 (17)	0.0542 (10)	
C25	0.8273 (4)	0.5199 (3)	0.66907 (18)	0.0664 (12)	
H25	0.8057	0.5717	0.6492	0.080*	
C26	0.7578 (4)	0.4342 (3)	0.57945 (18)	0.0682 (12)	
H26	0.7622	0.3720	0.5668	0.082*	
C27	0.6302 (5)	0.4608 (5)	0.5760 (3)	0.135 (3)	
H27A	0.5876	0.4291	0.6055	0.202*	
H27B	0.5980	0.4467	0.5376	0.202*	
H27C	0.6235	0.5235	0.5828	0.202*	
C28	0.8313 (7)	0.4877 (5)	0.5378 (2)	0.123 (2)	
H28A	0.8103	0.4723	0.4978	0.185*	
H28B	0.9132	0.4750	0.5451	0.185*	
H28C	0.8172	0.5499	0.5439	0.185*	
C29	0.9318 (5)	0.2826 (4)	0.7619 (2)	0.0933 (17)	
H29	0.9285	0.2336	0.7334	0.112*	
C30	0.8473 (7)	0.2609 (6)	0.8100 (4)	0.168 (4)	
H30A	0.8482	0.3080	0.8386	0.253*	
H30B	0.8707	0.2063	0.8288	0.253*	
H30C	0.7693	0.2546	0.7933	0.253*	
C31	1.0574 (6)	0.2868 (5)	0.7873 (3)	0.129 (2)	
H31A	1.0612	0.3293	0.8188	0.193*	
H31B	1.1101	0.3043	0.7569	0.193*	
H31C	1.0800	0.2293	0.8021	0.193*	
C32	0.7163 (4)	0.2424 (3)	0.64959 (16)	0.0545 (10)	
H32	0.6581	0.2726	0.6696	0.065*	
C33	0.6875 (3)	0.1580 (3)	0.62199 (15)	0.0487 (9)	
C34	0.7715 (3)	0.1114 (3)	0.58969 (17)	0.0523 (10)	
C35	0.7374 (4)	0.0327 (3)	0.56155 (18)	0.0582 (10)	
C36	0.6244 (4)	0.0016 (3)	0.56471 (18)	0.0599 (11)	
H36	0.6026	−0.0508	0.5456	0.072*	
C37	0.5435 (4)	0.0485 (3)	0.59632 (17)	0.0577 (11)	
C38	0.5731 (3)	0.1258 (3)	0.62503 (16)	0.0535 (10)	
H38	0.5171	0.1564	0.6464	0.064*	

### Atomic displacement parameters (Å^2^)

**Table:**

	*U*^11^	*U*^22^	*U*^33^	*U*^12^	*U*^13^	*U*^23^
Br1	0.1269 (5)	0.0750 (4)	0.0857 (4)	−0.0169 (3)	0.0053 (3)	0.0256 (3)
Br2	0.0774 (4)	0.0823 (4)	0.1261 (5)	0.0228 (3)	−0.0081 (3)	−0.0355 (3)
Cl1	0.0945 (9)	0.0948 (10)	0.0926 (9)	0.0458 (8)	−0.0318 (7)	−0.0111 (7)
Cl2	0.0632 (7)	0.0844 (8)	0.0938 (9)	−0.0237 (6)	−0.0056 (6)	0.0003 (7)
O1	0.0665 (18)	0.0584 (18)	0.080 (2)	0.0030 (14)	0.0035 (15)	0.0180 (15)
O2	0.0524 (17)	0.0645 (19)	0.084 (2)	−0.0023 (14)	−0.0048 (14)	−0.0114 (15)
N1	0.058 (2)	0.049 (2)	0.0534 (19)	0.0039 (16)	−0.0065 (15)	0.0063 (15)
N2	0.059 (2)	0.055 (2)	0.055 (2)	−0.0039 (16)	−0.0047 (16)	−0.0025 (16)
C1	0.071 (3)	0.052 (3)	0.071 (3)	0.000 (2)	0.011 (2)	0.009 (2)
C2	0.092 (3)	0.065 (3)	0.067 (3)	0.017 (3)	0.011 (3)	0.025 (3)
C3	0.093 (3)	0.079 (4)	0.053 (3)	0.015 (3)	−0.008 (2)	0.018 (3)
C4	0.073 (3)	0.065 (3)	0.048 (2)	0.008 (2)	−0.007 (2)	0.007 (2)
C5	0.050 (2)	0.048 (2)	0.051 (2)	0.0043 (18)	0.0009 (17)	0.0100 (19)
C6	0.050 (2)	0.054 (2)	0.050 (2)	0.0012 (18)	0.0025 (17)	0.006 (2)
C7	0.070 (3)	0.058 (3)	0.069 (3)	0.002 (2)	−0.009 (2)	−0.004 (2)
C8	0.102 (4)	0.190 (7)	0.064 (3)	0.030 (4)	0.000 (3)	−0.010 (4)
C9	0.068 (4)	0.263 (10)	0.104 (5)	−0.039 (5)	−0.011 (3)	−0.006 (5)
C10	0.109 (4)	0.084 (4)	0.072 (3)	0.007 (3)	−0.039 (3)	−0.003 (3)
C11	0.127 (6)	0.229 (10)	0.262 (11)	−0.018 (6)	0.058 (7)	−0.171 (9)
C12	0.104 (5)	0.158 (7)	0.146 (6)	−0.026 (4)	0.000 (4)	−0.046 (5)
C13	0.062 (3)	0.053 (2)	0.041 (2)	0.000 (2)	0.0007 (18)	0.0027 (18)
C14	0.065 (2)	0.045 (2)	0.038 (2)	0.0080 (19)	−0.0076 (18)	−0.0048 (17)
C15	0.072 (3)	0.043 (2)	0.041 (2)	0.003 (2)	−0.0069 (19)	−0.0025 (18)
C16	0.094 (3)	0.050 (3)	0.044 (2)	−0.001 (2)	−0.007 (2)	0.001 (2)
C17	0.109 (4)	0.049 (3)	0.052 (3)	0.018 (3)	−0.023 (3)	0.000 (2)
C18	0.083 (3)	0.059 (3)	0.054 (3)	0.023 (2)	−0.022 (2)	−0.007 (2)
C19	0.062 (2)	0.062 (3)	0.049 (2)	0.012 (2)	−0.0101 (19)	−0.008 (2)
C20	0.097 (4)	0.075 (3)	0.063 (3)	−0.025 (3)	0.005 (3)	−0.022 (3)
C21	0.094 (3)	0.087 (4)	0.050 (3)	−0.013 (3)	−0.009 (2)	−0.013 (3)
C22	0.077 (3)	0.072 (3)	0.052 (3)	−0.008 (2)	−0.004 (2)	−0.007 (2)
C23	0.053 (2)	0.057 (3)	0.052 (2)	−0.0062 (19)	0.0045 (19)	−0.011 (2)
C24	0.057 (2)	0.056 (3)	0.049 (2)	−0.0057 (19)	0.0018 (18)	−0.009 (2)
C25	0.080 (3)	0.059 (3)	0.060 (3)	−0.007 (2)	0.007 (2)	−0.007 (2)
C26	0.086 (3)	0.060 (3)	0.058 (3)	0.002 (2)	−0.014 (2)	−0.009 (2)
C27	0.100 (5)	0.185 (8)	0.117 (5)	0.036 (5)	−0.044 (4)	−0.016 (5)
C28	0.183 (6)	0.135 (5)	0.051 (3)	−0.051 (5)	−0.004 (3)	0.000 (3)
C29	0.125 (5)	0.091 (4)	0.062 (3)	−0.005 (3)	−0.023 (3)	0.004 (3)
C30	0.131 (6)	0.188 (8)	0.186 (8)	−0.012 (6)	0.008 (6)	0.109 (7)
C31	0.123 (5)	0.164 (7)	0.099 (5)	0.027 (5)	−0.016 (4)	0.031 (4)
C32	0.057 (3)	0.061 (3)	0.045 (2)	0.002 (2)	−0.0028 (18)	0.0005 (19)
C33	0.053 (2)	0.052 (2)	0.040 (2)	−0.0047 (18)	−0.0110 (17)	0.0057 (18)
C34	0.050 (2)	0.051 (2)	0.055 (2)	0.0019 (19)	−0.0112 (18)	0.007 (2)
C35	0.062 (3)	0.048 (2)	0.064 (3)	0.011 (2)	−0.015 (2)	0.001 (2)
C36	0.070 (3)	0.046 (2)	0.063 (3)	0.002 (2)	−0.017 (2)	0.007 (2)
C37	0.059 (2)	0.062 (3)	0.051 (2)	−0.011 (2)	−0.011 (2)	0.013 (2)
C38	0.056 (2)	0.057 (3)	0.047 (2)	−0.0009 (19)	−0.0038 (18)	0.0064 (19)

### Geometric parameters (Å, °)

**Table:**

Br1—C16	1.880 (5)	C15—C16	1.388 (5)
Br2—C35	1.878 (4)	C16—C17	1.392 (6)
Cl1—C18	1.748 (5)	C17—C18	1.369 (6)
Cl2—C37	1.751 (4)	C17—H17	0.9300
O1—C15	1.339 (5)	C18—C19	1.351 (6)
O1—H1A	0.8200	C19—H19	0.9300
O2—C34	1.339 (4)	C20—C21	1.368 (7)
O2—H2A	0.8200	C20—C25	1.382 (6)
N1—C13	1.273 (5)	C20—H20	0.9300
N1—C5	1.427 (5)	C21—C22	1.387 (6)
N2—C32	1.276 (5)	C21—H21	0.9300
N2—C23	1.434 (5)	C22—C23	1.406 (5)
C1—C2	1.377 (6)	C22—C29	1.503 (7)
C1—C6	1.389 (5)	C23—C24	1.390 (5)
C1—H1	0.9300	C24—C25	1.381 (6)
C2—C3	1.370 (7)	C24—C26	1.523 (5)
C2—H2	0.9300	C25—H25	0.9300
C3—C4	1.380 (6)	C26—C27	1.504 (7)
C3—H3	0.9300	C26—C28	1.507 (7)
C4—C5	1.402 (5)	C26—H26	0.9800
C4—C10	1.510 (6)	C27—H27A	0.9600
C5—C6	1.393 (5)	C27—H27B	0.9600
C6—C7	1.521 (5)	C27—H27C	0.9600
C7—C9	1.503 (7)	C28—H28A	0.9600
C7—C8	1.507 (7)	C28—H28B	0.9600
C7—H7	0.9800	C28—H28C	0.9600
C8—H8A	0.9600	C29—C30	1.505 (8)
C8—H8B	0.9600	C29—C31	1.527 (8)
C8—H8C	0.9600	C29—H29	0.9800
C9—H9A	0.9600	C30—H30A	0.9600
C9—H9B	0.9600	C30—H30B	0.9600
C9—H9C	0.9600	C30—H30C	0.9600
C10—C11	1.482 (8)	C31—H31A	0.9600
C10—C12	1.522 (8)	C31—H31B	0.9600
C10—H10	0.9800	C31—H31C	0.9600
C11—H11A	0.9600	C32—C33	1.450 (5)
C11—H11B	0.9600	C32—H32	0.9300
C11—H11C	0.9600	C33—C38	1.390 (5)
C12—H12A	0.9600	C33—C34	1.404 (5)
C12—H12B	0.9600	C34—C35	1.395 (5)
C12—H12C	0.9600	C35—C36	1.371 (6)
C13—C14	1.448 (5)	C36—C37	1.373 (6)
C13—H13	0.9300	C36—H36	0.9300
C14—C19	1.398 (5)	C37—C38	1.370 (5)
C14—C15	1.403 (5)	C38—H38	0.9300
			
C15—O1—H1A	109.5	C18—C19—H19	119.7
C34—O2—H2A	109.5	C14—C19—H19	119.7
C13—N1—C5	119.9 (3)	C21—C20—C25	119.7 (4)
C32—N2—C23	118.7 (3)	C21—C20—H20	120.1
C2—C1—C6	120.7 (4)	C25—C20—H20	120.1
C2—C1—H1	119.7	C20—C21—C22	122.2 (4)
C6—C1—H1	119.7	C20—C21—H21	118.9
C3—C2—C1	120.7 (4)	C22—C21—H21	118.9
C3—C2—H2	119.7	C21—C22—C23	116.5 (4)
C1—C2—H2	119.7	C21—C22—C29	121.8 (4)
C2—C3—C4	121.4 (4)	C23—C22—C29	121.6 (4)
C2—C3—H3	119.3	C24—C23—C22	122.3 (4)
C4—C3—H3	119.3	C24—C23—N2	119.5 (3)
C3—C4—C5	117.0 (4)	C22—C23—N2	118.1 (4)
C3—C4—C10	121.7 (4)	C25—C24—C23	118.1 (4)
C5—C4—C10	121.3 (4)	C25—C24—C26	120.0 (4)
C6—C5—C4	122.8 (4)	C23—C24—C26	121.8 (4)
C6—C5—N1	119.6 (3)	C24—C25—C20	120.9 (4)
C4—C5—N1	117.5 (4)	C24—C25—H25	119.6
C1—C6—C5	117.3 (4)	C20—C25—H25	119.6
C1—C6—C7	119.6 (4)	C27—C26—C28	111.9 (5)
C5—C6—C7	123.0 (4)	C27—C26—C24	112.3 (4)
C9—C7—C8	111.1 (5)	C28—C26—C24	111.1 (4)
C9—C7—C6	113.3 (4)	C27—C26—H26	107.1
C8—C7—C6	111.0 (4)	C28—C26—H26	107.1
C9—C7—H7	107.0	C24—C26—H26	107.1
C8—C7—H7	107.0	C26—C27—H27A	109.5
C6—C7—H7	107.0	C26—C27—H27B	109.5
C7—C8—H8A	109.5	H27A—C27—H27B	109.5
C7—C8—H8B	109.5	C26—C27—H27C	109.5
H8A—C8—H8B	109.5	H27A—C27—H27C	109.5
C7—C8—H8C	109.5	H27B—C27—H27C	109.5
H8A—C8—H8C	109.5	C26—C28—H28A	109.5
H8B—C8—H8C	109.5	C26—C28—H28B	109.5
C7—C9—H9A	109.5	H28A—C28—H28B	109.5
C7—C9—H9B	109.5	C26—C28—H28C	109.5
H9A—C9—H9B	109.5	H28A—C28—H28C	109.5
C7—C9—H9C	109.5	H28B—C28—H28C	109.5
H9A—C9—H9C	109.5	C22—C29—C30	110.8 (5)
H9B—C9—H9C	109.5	C22—C29—C31	113.4 (5)
C11—C10—C4	110.7 (5)	C30—C29—C31	110.0 (5)
C11—C10—C12	109.6 (5)	C22—C29—H29	107.4
C4—C10—C12	113.6 (5)	C30—C29—H29	107.4
C11—C10—H10	107.6	C31—C29—H29	107.4
C4—C10—H10	107.6	C29—C30—H30A	109.5
C12—C10—H10	107.6	C29—C30—H30B	109.5
C10—C11—H11A	109.5	H30A—C30—H30B	109.5
C10—C11—H11B	109.5	C29—C30—H30C	109.5
H11A—C11—H11B	109.5	H30A—C30—H30C	109.5
C10—C11—H11C	109.5	H30B—C30—H30C	109.5
H11A—C11—H11C	109.5	C29—C31—H31A	109.5
H11B—C11—H11C	109.5	C29—C31—H31B	109.5
C10—C12—H12A	109.5	H31A—C31—H31B	109.5
C10—C12—H12B	109.5	C29—C31—H31C	109.5
H12A—C12—H12B	109.5	H31A—C31—H31C	109.5
C10—C12—H12C	109.5	H31B—C31—H31C	109.5
H12A—C12—H12C	109.5	N2—C32—C33	122.3 (4)
H12B—C12—H12C	109.5	N2—C32—H32	118.8
N1—C13—C14	121.9 (4)	C33—C32—H32	118.8
N1—C13—H13	119.1	C38—C33—C34	120.0 (4)
C14—C13—H13	119.1	C38—C33—C32	119.1 (4)
C19—C14—C15	119.3 (4)	C34—C33—C32	120.8 (3)
C19—C14—C13	119.5 (4)	O2—C34—C35	119.1 (4)
C15—C14—C13	121.2 (3)	O2—C34—C33	122.4 (3)
O1—C15—C16	119.5 (4)	C35—C34—C33	118.5 (4)
O1—C15—C14	122.0 (3)	C36—C35—C34	121.1 (4)
C16—C15—C14	118.5 (4)	C36—C35—Br2	120.3 (3)
C15—C16—C17	121.2 (4)	C34—C35—Br2	118.5 (3)
C15—C16—Br1	118.4 (3)	C35—C36—C37	119.4 (4)
C17—C16—Br1	120.4 (3)	C35—C36—H36	120.3
C18—C17—C16	118.8 (4)	C37—C36—H36	120.3
C18—C17—H17	120.6	C38—C37—C36	121.6 (4)
C16—C17—H17	120.6	C38—C37—Cl2	119.0 (3)
C19—C18—C17	121.6 (4)	C36—C37—Cl2	119.4 (3)
C19—C18—Cl1	119.9 (4)	C37—C38—C33	119.4 (4)
C17—C18—Cl1	118.5 (3)	C37—C38—H38	120.3
C18—C19—C14	120.6 (4)	C33—C38—H38	120.3
			
C6—C1—C2—C3	−1.5 (7)	C25—C20—C21—C22	−1.8 (8)
C1—C2—C3—C4	2.1 (7)	C20—C21—C22—C23	−0.9 (7)
C2—C3—C4—C5	−0.1 (7)	C20—C21—C22—C29	−179.7 (5)
C2—C3—C4—C10	179.0 (5)	C21—C22—C23—C24	4.1 (6)
C3—C4—C5—C6	−2.6 (6)	C29—C22—C23—C24	−177.0 (4)
C10—C4—C5—C6	178.4 (4)	C21—C22—C23—N2	−179.7 (4)
C3—C4—C5—N1	−179.0 (4)	C29—C22—C23—N2	−0.9 (6)
C10—C4—C5—N1	2.0 (6)	C32—N2—C23—C24	−82.8 (5)
C13—N1—C5—C6	71.3 (5)	C32—N2—C23—C22	100.9 (4)
C13—N1—C5—C4	−112.2 (4)	C22—C23—C24—C25	−4.6 (6)
C2—C1—C6—C5	−1.0 (6)	N2—C23—C24—C25	179.3 (3)
C2—C1—C6—C7	176.6 (4)	C22—C23—C24—C26	174.5 (4)
C4—C5—C6—C1	3.1 (6)	N2—C23—C24—C26	−1.7 (6)
N1—C5—C6—C1	179.4 (3)	C23—C24—C25—C20	1.8 (6)
C4—C5—C6—C7	−174.4 (4)	C26—C24—C25—C20	−177.3 (4)
N1—C5—C6—C7	2.0 (6)	C21—C20—C25—C24	1.3 (7)
C1—C6—C7—C9	60.1 (6)	C25—C24—C26—C27	−70.8 (6)
C5—C6—C7—C9	−122.5 (5)	C23—C24—C26—C27	110.2 (5)
C1—C6—C7—C8	−65.7 (6)	C25—C24—C26—C28	55.4 (6)
C5—C6—C7—C8	111.7 (5)	C23—C24—C26—C28	−123.7 (5)
C3—C4—C10—C11	−74.4 (7)	C21—C22—C29—C30	75.9 (7)
C5—C4—C10—C11	104.6 (6)	C23—C22—C29—C30	−102.9 (6)
C3—C4—C10—C12	49.4 (7)	C21—C22—C29—C31	−48.4 (7)
C5—C4—C10—C12	−131.6 (5)	C23—C22—C29—C31	132.9 (5)
C5—N1—C13—C14	−176.3 (3)	C23—N2—C32—C33	177.4 (3)
N1—C13—C14—C19	177.5 (4)	N2—C32—C33—C38	−178.4 (4)
N1—C13—C14—C15	0.3 (5)	N2—C32—C33—C34	−2.0 (6)
C19—C14—C15—O1	−178.6 (3)	C38—C33—C34—O2	178.5 (3)
C13—C14—C15—O1	−1.4 (5)	C32—C33—C34—O2	2.2 (5)
C19—C14—C15—C16	0.8 (5)	C38—C33—C34—C35	−0.5 (5)
C13—C14—C15—C16	178.1 (3)	C32—C33—C34—C35	−176.8 (3)
O1—C15—C16—C17	179.2 (4)	O2—C34—C35—C36	−178.4 (4)
C14—C15—C16—C17	−0.3 (6)	C33—C34—C35—C36	0.6 (6)
O1—C15—C16—Br1	0.3 (5)	O2—C34—C35—Br2	−0.3 (5)
C14—C15—C16—Br1	−179.2 (3)	C33—C34—C35—Br2	178.7 (3)
C15—C16—C17—C18	−0.5 (6)	C34—C35—C36—C37	−0.3 (6)
Br1—C16—C17—C18	178.3 (3)	Br2—C35—C36—C37	−178.3 (3)
C16—C17—C18—C19	0.8 (6)	C35—C36—C37—C38	−0.2 (6)
C16—C17—C18—Cl1	179.3 (3)	C35—C36—C37—Cl2	179.8 (3)
C17—C18—C19—C14	−0.3 (6)	C36—C37—C38—C33	0.3 (6)
Cl1—C18—C19—C14	−178.7 (3)	Cl2—C37—C38—C33	−179.6 (3)
C15—C14—C19—C18	−0.5 (6)	C34—C33—C38—C37	0.0 (5)
C13—C14—C19—C18	−177.8 (3)	C32—C33—C38—C37	176.4 (3)

### Hydrogen-bond geometry (Å, °)

**Table:**

*D*—H···*A*	*D*—H	H···*A*	*D*···*A*	*D*—H···*A*
O1—H1A···N1	0.82	1.87	2.598 (4)	147
O2—H2A···N2	0.82	1.88	2.610 (4)	147
C28—H28A···Cg1^i^	0.96	2.96	3.773 (6)	144
C16—Br1···Cg4^i^	1.880	3.53	4.75 (2)	120

Symmetry codes: (i) −*x*+1, −*y*+1, −*z*+1.

## References

[bb1] Bruker (2004). APEX2 and SAINT. Bruker AXS Inc., Madison, Wisconsin, USA.

[bb2] Chang, S., Jones, L. II, Wang, C., Henling, L. M. & Grubbs, R. H. (1998). *Organometallics*, **17**, 3460–3465.

[bb3] Chen, F. & Ye, H.-Y. (2008). *Acta Cryst.* E**64**, o1757. 10.1107/S160053680802552X PMC2960556 21201739

[bb4] Farrugia, L. J. (1997). *J. Appl. Cryst.* **30**, 565.

[bb5] Figuet, M., Averbuch-Pouchot, M. T., du Moulinet d’Hardemare, A. & Jarjayes, O. (2001). *Eur. J. Inorg. Chem.* pp. 2089–2096.

[bb6] Kennedy, A. R. & Reglinski, J. (2001). *Acta Cryst.* E**57**, o1027–o1028.

[bb7] Lin, J., Cui, G.-H., Li, J.-R. & Xu, S.-S. (2005). *Acta Cryst.* E**61**, o627–o628.

[bb8] Pu, X.-H. (2008). *Acta Cryst.* E**64**, o1734. 10.1107/S1600536808024884 PMC2960476 21201717

[bb9] Sheldrick, G. M. (1996). *SADABS* University of Göttingen, Germany.

[bb10] Sheldrick, G. M. (2008). *Acta Cryst.* A**64**, 112–122. 10.1107/S0108767307043930 18156677

[bb11] Spek, A. L. (2003). *J. Appl. Cryst.* **36**, 7–13.

[bb12] Thamotharan, S., Parthasarathi, V., Anitha, S. M., Prasad, A., Rao, T. R. & Linden, A. (2003). *Acta Cryst.* E**59**, o1856–o1857.

